# Study on the diagnostic value of folic acid-targeted nano-microbubbles in breast microcarcinoma

**DOI:** 10.3389/fonc.2025.1512422

**Published:** 2025-07-09

**Authors:** Nianyu Xue, Zhenyu Shen, Zhenbin Xu, Shengmin Zhang

**Affiliations:** Department of Ultrasonography, The First Affiliated Hospital of Ningbo University, Ningbo First Hospital, Ningbo, Zhejiang, China

**Keywords:** folic acid, breast cancer, microbubble, ultrasonography, diagnosis

## Abstract

**Background:**

The prognosis of breast cancer is significantly correlated with its early detection. It is difficult to detect breast microcarcinomas less than or equal to 5 mm by imaging examination.

**Methods:**

Folic acid-targeted nanobubbles (FA-TNBs) were prepared by the thin-film hydration method. Cytotoxicity and cellular uptake assays were used to examine FA-TNBs’ biological toxicity to cells, targeting them to breast cancer cells. In addition, by constructing 4T1 tumor mouse models and evaluating the targeting and imaging effects of FA-TNBs.

**Results:**

The average particle size of the fabricated FA-TNBs was 244 ± 21 nm. *In vitro*, cell uptake experiments showed that breast cancer 4T1 cells take up more FA-TNBs than non-targeted nanobubbles (N-TNBs) (p < 0.001). In the cytotoxicity experiment, the survival rate of 4T1 cells under each FA-TNBs concentration was over 90%. *In vivo*, imaging of the mouse 4T1 tumor model showed that compared with the N-TNBs group, the FA-TNBs group took a shorter time to peak (20 s, 40 s, p < 0.05), had a higher peak intensity (38.3 ± 1.5 dB, 31.7 ± 1.5 dB, p < 0.05), and the extinction time was shorter (180 s, 120 s, p < 0.05). After FA-TNB injection, there were no apparent abnormalities in the tissue sections or hematological examinations of the mice’s vital organs.

**Conclusions:**

The prepared FA-TNBs had good water solubility, safety, biocompatibility, and enhancement of ultrasound imaging. It had an excellent imaging effect on mouse breast cancer tumors with a diameter of 5 mm and showed apparent active targeting. FA-TNBs may become a new and practical ultrasound contrast agent for the early detection of breast cancer.

## Introduction

Breast cancer has become the most common malignant tumor in women, accounting for about 30% of female cancers (1). The prognosis of breast cancer is significantly correlated with its early detection. Early breast cancer has a higher survival rate than advanced disease, and it allows for breast-conserving surgery to be performed (2). Although imaging (including magnetic resonance, mammography, and ultrasonography) plays a significant role in breast cancer detection, it remains challenging to detect breast cancers less than or equal to 5 mm with this method of examination (3).

Currently, the ultrasound contrast agent most often in clinical use only flows within the blood vessels and does not enter the interstitial space. Due to a lack of targeting, the identification of breast microcarcinoma is poor (4, 5). The further improvement of clinical breast microcarcinoma diagnosis has become problematic. In recent years, targeting nano-microbubbles(nanobubbles) has become a research focus in the early diagnosis of tumors (6, 7).

Studies have shown that the positive expression rate of folate receptor alpha (FR-α) in breast cancer is relatively high (8). In addition, FR-α expressed in malignant tumor tissues retains its cell surface distribution and ability to bind folic acid. These features allow for the use of tumor cell FR-α as a selectively targeted imaging agent fabrication method (9, 10).

Currently, targeted contrast agents are primarily used to focus on tumor blood vessels for benign and malignant identification and treatment (11). In contrast, malignant tumors do not depend on neovascularization when they do not exceed 5 mm (12). Therefore, we chose liposome nanobubbles combined with folic acid to make targeted nanobubbles. We aimed to study their diagnostic value in breast microcarcinoma (tumor diameter less than or equal to 5 mm). Hypothesis: Folic acid-modified targeted nanobubbles (FA-TNBs) can improve the ultrasound imaging of breast microcarcinoma through enhanced tumor-specific accumulation, thereby offering greater diagnostic sensitivity compared to non-targeted nanobubbles. Primary Objective: To develop and evaluate the imaging efficacy and biosafety of FA-TNBs as a targeted ultrasound contrast agent for the early detection of breast microcarcinoma *in vitro* and *in vivo*.

## Materials and methods

### Materials

DSPE-PEG(2000), DSPE-PEG(2000)-FA, and DSPE-PEG(2000)-FICT were purchased from Aiwei Tuo (Shanghai) Co., Ltd., and C3H8 gas was purchased from Wula (Hangzhou) Chemical Co., Ltd. In addition, chloroform, agar powder, sodium chloride, potassium chloride, and dipotassium hydrogen phosphate were purchased from Sinopharm Chemical Reagent Co., Ltd. DMEM (high glucose), the cell culture medium, and fetal bovine serum were purchased from Gibco (USA). Furthermore, dimethyl sulfoxide, penicillin-streptomycin double antibody, the Dil staining kit, the CCK-8 kit, and DAPI dye were purchased from Biyuntian Biotechnology Co., Ltd. Lastly, 0.25% trypsin and PBS7.4 solution were purchased from Biosharp Saiguo Biotechnology Company.

### Cells and animals

The 4T1 mouse breast cancer cells were provided by the Shanghai Chinese Academy of Sciences Cell Bank, and the culture medium was configured as DMEM high glucose medium + fetal bovine serum + penicillin-streptomycin double-antibody. Fifteen female Balb/c mice aged 6–8 weeks (provided by Viton Lever Laboratory Animal Center) were reared for one week before the experiment to a body weight of about 16–18 g. The animal experiments were approved by the Association of Animal Experiment Ethics and Ethics of Ningbo University.

### Preparation of nanobubbles

Liposome nanobubbles were prepared by the thin film hydration method. The exact amounts of each component used for nanobubble preparation are as follows: DPPC: 10 mg, DSPE-PEG-FA(2000): 4 mg, Cholesterol: 1 mg. These components were dissolved in 3 mL of chloroform according to a mass ratio of 10:4:1 (DPPC: DSPE-PEG-FA(2000):cholesterol). After solvent evaporation, the lipid film was hydrated with PBS to form liposomes, followed by perfluoropropane gas encapsulation. Perfluoropropane (C_3_F_8_) gas was introduced during the nanobubble preparation process by replacing the air in the headspace of the vial with C_3_F_8_ gas under sterile conditions. After hydration of the lipid film with PBS, the vial was sealed, and the air inside was evacuated using a syringe before being filled with perfluoropropane gas. The vial was then vigorously shaken using a mechanical vibrator or vortex mixer for 1 min to facilitate gas encapsulation and nanobubble formation. Then, the organic solvent was rotary evaporated to form a lipid film on the bottom of an eggplant-shaped bottle, which was hydrated after cooling to room temperature and continuously mixed. During rotary evaporation, the pressure was reduced to approximately 150 mmHg below atmospheric pressure, and evaporation was performed at 45°C for 30 minutes to ensure thorough solvent removal. The resulting emulsion was further processed using an ultrasonic cell disintegrator before being filled with perfluoropropane gas. Ultrasonic disintegration was carried out using a probe sonicator at a power of 30 W, operating in cycles of 2 seconds on and 2 seconds off, with a total sonication time of 5 minutes to avoid overheating while ensuring effective dispersion. It was rapidly oscillated using an amalgam blender to obtain nanobubbles, which were stored in a 4°C refrigerator. All these operations were conducted away from the light. The folic acid-targeted nanobubbles (FA-TNBs) were prepared by adding 4 mg DSPE-PEG-FA in the first step, with the remaining steps continuing as above. Finally, non-targeted nanobubbles (N-TNBs) and FA-TNBs were obtained.

Before measurement, FA-TNBs were diluted using phosphate-buffered saline (PBS, pH 7.4) as the diluent. The standard dilution factor was 1:100 (v/v). The FA-TNBs were diluted to an appropriate concentration and measured on a nanoparticle-sized Zeta potential analyzer (Nano-ZS, Malvern, England) to obtain the particle size, dispersion coefficient, and Zeta potential. Lastly, the FA-TNB and N-TNB morphologies were detected via scanning electron microscopy (SEM, S4800, Hitachi, Tokyo, Japan).

### Cell culture

The 4T1 murine breast cancer cell line used in our experiments was obtained from the Cell Bank of the Chinese Academy of Sciences (Shanghai, China). Cells were used between passages 3 and 10 from the original stock to ensure biological consistency and minimize phenotypic drift during the experiments. The 4T1 cells were cultured on a DMEM high-glucose medium containing 10% FBS and 1% penicillin-streptomycin and placed in an incubator (temperature: 37°C, CO2 volume fraction: 5%) to promote cell growth. The cell culture media were replaced every two to three days, depending on the growth of the tumor cells.

### Cytotoxicity and uptake assays

Cytotoxicity was tested by the CCK-8 method. The 4T1 cells grown during the logarithmic growth phase were cultured on a 96-well plates with 100 uL added to each well. The culture plates were incubated in a constant-temperature cell incubator at 37°C for 24 hours, and the culture medium was then aspirated. Add 100 ul of FA-TNB-containing culture medium to each well, incubate for 24 hours, and add 10 ul of CCK-8 to each well. Then, the culture medium was incubated at 37°C for one to two hours, and the viability of 4T1 cells was evaluated using a microplate reader.

Next, the Dil-labeled FA-TNBs and NTNBs were prepared. In a 6-well plate, 2 mL of complete DMEM culture medium containing 4T1 cells was inoculated into each well and incubated for 24 hours in a constant temperature cell incubator at 37°C. Then, the culture medium was aspirated, and washed twice with 1 mL of PBS solution, and an appropriate amount of 4% paraformaldehyde (PFA) was added to cover the bottom of the cells. The plate was incubated for 15 min in a constant temperature cell incubator, the 4% PFA was aspirated, and the medium was washed twice with PBS solution.

Add the right amount of DAPI staining solution to each well to cover the bottom; Place the plate in an incubator for 15 minutes, remove them, and wash them twice with PBS solution. Then, DMEM medium containing a certain amount of FA-TNBs and NTBs was added to the six-well plate, and the process was repeated three times. For the cytotoxicity assay, FA-NBs were prepared with different concentrations of DPPC, and these varying formulations were used to assess toxicity. The DPPC concentrations tested were 2, 4, 6, 8, and 10 mg/mL. Cells were incubated with FA-NBs containing these different DPPC concentrations, and cell viability was evaluated after 24 hours using the CCK-8 assay. This range allowed assessment of cytotoxicity across formulations with increasing lipid content, providing insight into the safety profile of FA-NBs with varying DPPC levels. After being placed in a constant temperature incubator for four hours, the culture medium was aspirated and washed three times with PBS solution. Finally, a small amount of PBS solution was added to the wells for easy observation.

The FA-TNBs-Dil or NTBs-Dil entered the 4T1 cells or combined with the cell membrane. The nucleus presented blue under the FV-1000 confocal microscope (Japan), with 358 nm excitation light. However, FA-TNBs-Dil or NTBs-Dil appeared orange-red with an excitation light of 549 nm.

To further validate the cellular uptake experiments, we performed flow cytometry to obtain quantitative data for comparison. The experimental steps were as follows: fabrication of FA-NTBs and NTBs was conducted with FICT fluorescent dyes. In the flow cytometry experiments, FITC fluorescence was detected using a standard filter set with an excitation wavelength of 488 nm (from the blue laser) and an emission filter centered around 530 ± 15 nm (commonly a 530/30 nm bandpass filter). This filter configuration effectively captures the green fluorescence emitted by FITC-labeled samples, ensuring optimal sensitivity and specificity in detecting the labeled nanobubbles during cell uptake analysis. In a 12-well plate, 1 mL of DMEM medium containing 4T1 was inoculated into each well and incubated in a constant temperature cell incubator at 37°C for 24 hours. Then, the culture medium was aspirated, washed twice with PBS solution, and 1 mL of DMEM medium containing a certain amount of FA-NTBs-FICT or NTBs-FICT was added and incubated with the cells for 12 hours. Finally, three wells of cells were set as a blank group, and only ordinary DMEM medium without microvesicles was added.

After adding 0.5 mL of trypsin for 7 minutes, 1 mL of culture medium was added, and the mixture was placed in a centrifuge tube for centrifugation (1500 rpm/min, 3 min). Then, the supernatant was discarded, and the cells were remixed with 0.5 mL of PBS solution. Flow cytometry determined the number of nanovesicles entering the cells by measuring the FA-NTBs-FICT or NTBs-FICT.

### *In vitro* imaging

The specific concentration of the FA-TNBs suspension in PBS for *in vitro* imaging was typically prepared using a DPPC concentration of 5 mg/mL, which provides optimal ultrasound contrast and stability. To create the holes in the agar block, before the agar powder and water mixture was heated, dissolved, and cooled to solidify, 4 mL Eppendorf tubes (EP tubes) were inserted into the molten agar. Once the agar solidified, the tubes were carefully removed, leaving uniform and precise holes. The FA-TNBs suspension was then injected into these pre-formed holes using a microsyringe with a fine needle, ensuring accurate placement without disrupting the agar structure. The nano-microbubbles were prepared in a specific concentration of PBS suspension with a pH of 7.4, injected into the holes of the agar block, and ultrasonic imaging was performed using a Mindray M9 portable ultrasonic imager. The ultrasonic signals of the PBS buffer and FA-NTBs contrast agent in the agar block holes were tested, and the enhancement intensity of the contrast agent was quantitatively analyzed.

### *In vivo* imaging experiments in animals

Murine breast cancer 4T1 cells were selected to construct this experimental animal model. In the animal model, tumor inoculation was performed by injecting 1×10^6 4T1 cells suspended in 150 µL of sterile PBS. The injection site was located on the dorsal area above the right thigh of each mouse. Using a 27-gauge needle, the cell suspension was injected subcutaneously at a depth of approximately 2–3 mm. The injection was carried out slowly over about 10 seconds to ensure even cell distribution and minimize tissue trauma. After injection, gentle pressure was applied with sterile gauze to prevent leakage and promote retention of the cells at the inoculation site. Tumor growth in the mice was observed daily, and tumor size was measured using digital calipers.

After the tumor on the right mouse thigh grew to a diameter of about 5 mm, six similar mice were selected and divided into two groups. A total of 200 ul of FA-TNBs and N-TNBs were injected intratumorally, and the M9 (Minray, China) portable ultrasound machine (L12 - 4S linear array probe, frequency: 4–12 MHZ, mechanical index: 0.12) was used for intertumoral injection. First, the tumor surface was gently touched for CEUS imaging. During imaging procedures, mice were anesthetized using isoflurane inhalation anesthesia. Specifically, mice were placed in an induction chamber with 3% isoflurane in oxygen for induction, followed by maintenance anesthesia with 1.5–2% isoflurane delivered via a nose cone. This method ensured rapid induction and easy control of anesthesia depth while minimizing stress and discomfort to the animals throughout the imaging session. For *in vivo* contrast-enhanced ultrasound (CEUS) imaging, the ultrasound probe was carefully positioned to make gentle contact with the skin over the tumor site without applying excessive pressure. To avoid disrupting the tumor tissue or affecting the distribution of nanobubbles, light and steady contact was maintained throughout the imaging process. The operator ensured minimal movement and avoided compressing the tumor by using a coupling gel to facilitate probe-skin contact and by stabilizing the probe with a mechanical holder when possible. This approach preserved the tumor’s natural state, allowing accurate and consistent CEUS imaging results. Images were collected at 10, 20, 30, 120, 180, and 300 s after injection. Then, 200 ul of NTBs and FA-TNBs were injected from the tail vein of each mouse for contrast-enhanced ultrasound imaging. The other methods were the same as above.

### Biosafety testing

Six healthy BLab/c mice were selected and randomly divided into a blank control group and a nano-microbubble group. PBS buffer and FA-TNBs 200 ul were injected into the tail vein, respectively. After two weeks of regular feeding, the mice were sacrificed, and organ tissue section analysis was performed on five principal organs (heart, liver, spleen, lung, and kidney). For organ tissue section analysis, harvested tissues were first fixed in 10% neutral buffered formalin for 24 hours at room temperature to preserve cellular morphology. Following fixation, tissues were dehydrated through a graded ethanol series, cleared with xylene, and embedded in paraffin. Thin sections of approximately 4–5 µm thickness were then cut using a microtome and mounted onto glass slides. The tissue sections were subsequently deparaffinized, rehydrated, and stained with hematoxylin and eosin (H&E) following standard protocols to evaluate histopathological changes. After staining, slides were dehydrated, cleared, and coverslipped for microscopic examination. In addition, the blood routine of the mice was analyzed simultaneously. The blood routine analysis included measurement of the following parameters: white blood cell (WBC) count, red blood cell (RBC) count, hemoglobin (HGB) concentration, hematocrit (HCT), platelet (PLT) count, mean corpuscular volume (MCV), mean corpuscular hemoglobin (MCH), and mean corpuscular hemoglobin concentration (MCHC). These parameters were assessed to evaluate the general hematological health and detect any potential hematotoxicity associated with the treatment.

### Statistical analysis

The experimental data were analyzed by SPSS 18.0 statistics. SPSS 18.0 provides reliable functions for performing descriptive statistics, t-tests, ANOVA, and non-parametric tests suitable for the data type and sample size in this study. Comparison of cell channel targeting and FICT fluorescence values in cells among the three groups, FA-TNBs group, N-TNBs group, and blank control group, was performed by one-way ANOVA-test. Comparison of peak intensity and fading time after contrast agent injection into nude mice between FA-TNBs and N-TNBs groups was performed using two-sample independent t-test. Notably, p < 0.05 was considered statistically significant.

## Results

### Characterization of nanobubbles

**Figure 1:**
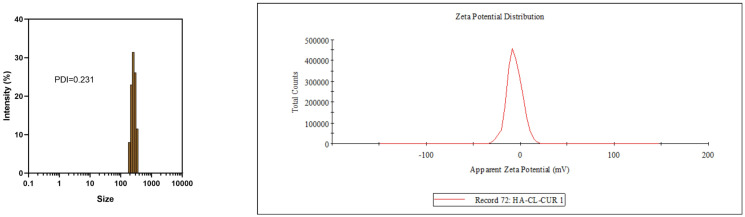
Particle size and potential diagram of FA-TNBs.

**Figure 2:**
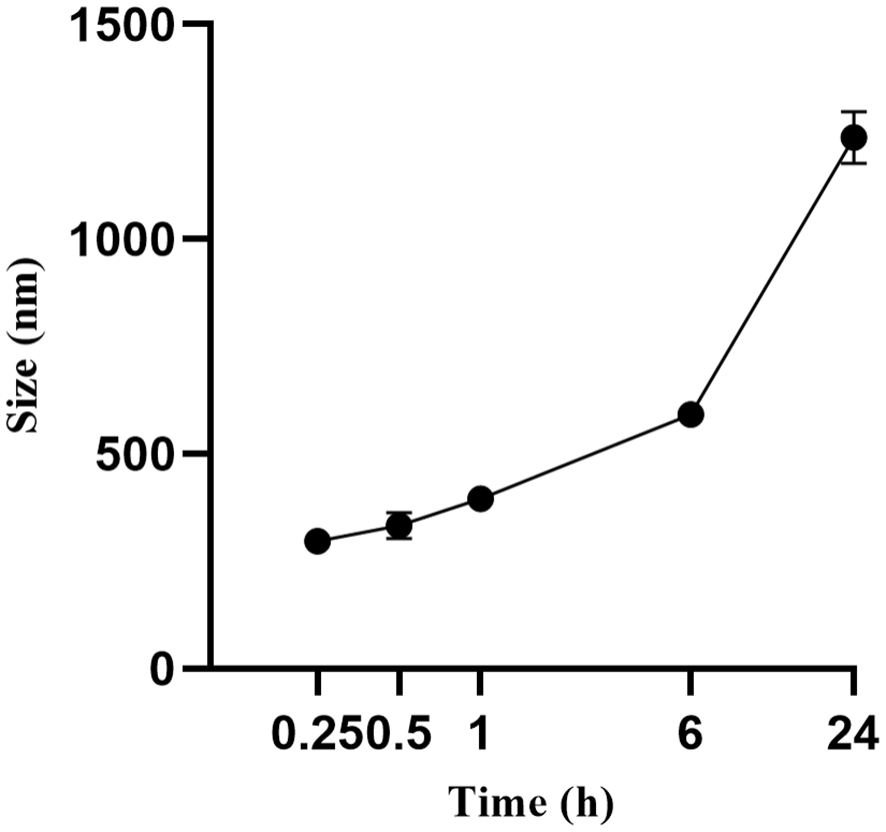
Change of FA-TNBs particle size after standing for 24 hours.

The FA-TNB particle size was about 244 ± 21 nm (n = 3). The PDI was 0.28 ± 0.07 (n = 3). In addition, the zeta potential was 6.04 ± 0.67 mv (n = 3) (**Figure 1**). After standing for 10 mins, 30 mins, and one hour, the particle size increased slightly, but the differences were not significant. With the increase in particle size after six hours, the PDI also increased significantly. When measured again after standing for 24 h, the particle size increased significantly (**Figure 2**). Therefore, to ensure consistency and reliability, all nanobubble preparations used in our experiments were freshly prepared immediately prior to use.

**Figure 3:**
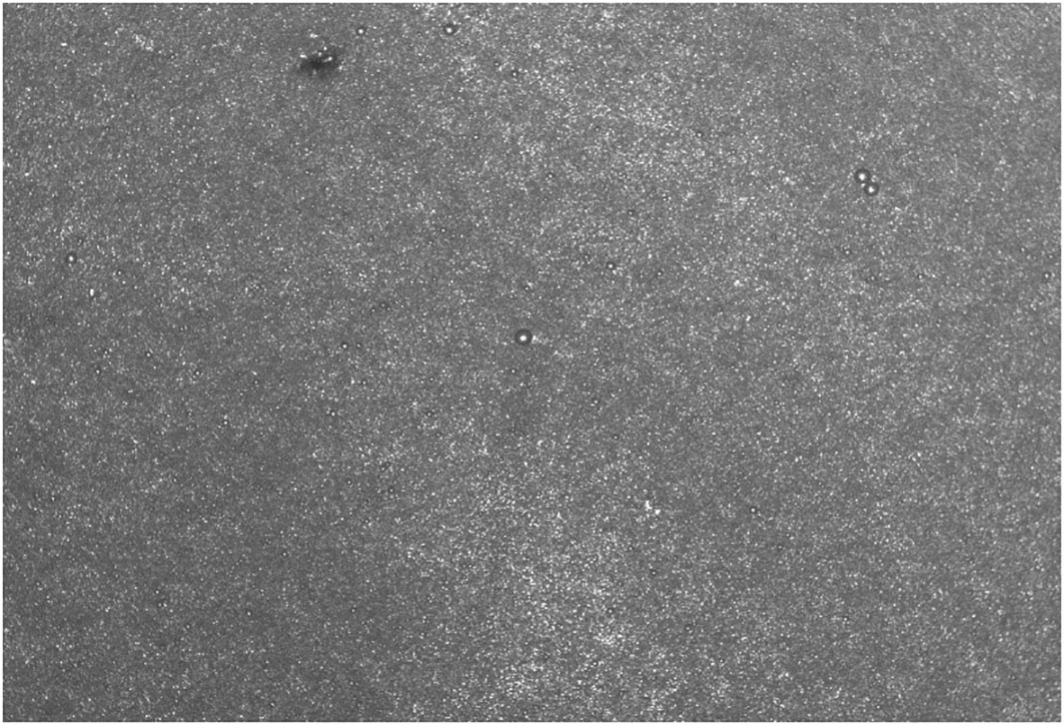
FA-TNBs diagram under automatic cell counter (Thermo Fisher).

An automated cell counter showed a microvesicle concentration of approximately 1*10^7/mL (**Figure 3**).

**Figure 4:**
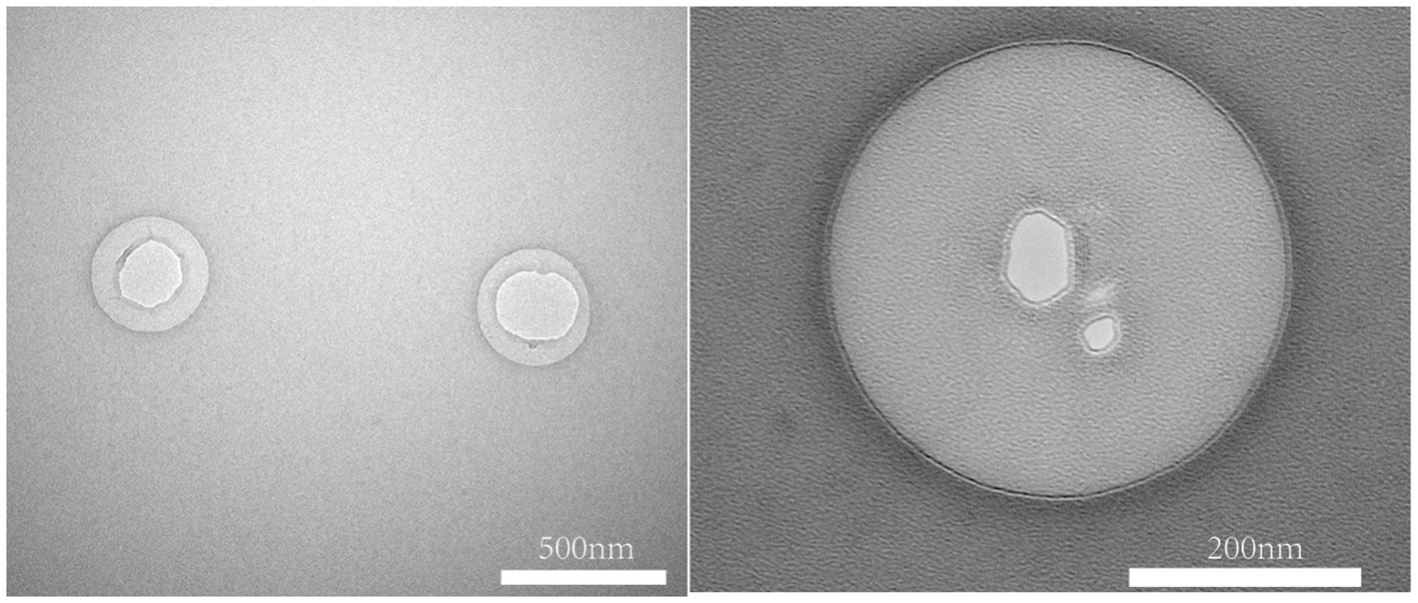
Image under FA-NBs Transmission Electron Microscopy (TEM).

The TEM images showed that the FA-TNB morphology was almost spherical and well-dispersed in the solution. A slightly brighter gas filled the liposomes (**Figure 4**). Notably, most FA-TNBs were below 500 nm in diameter.

### Cell uptake assay

**Figure 5:**
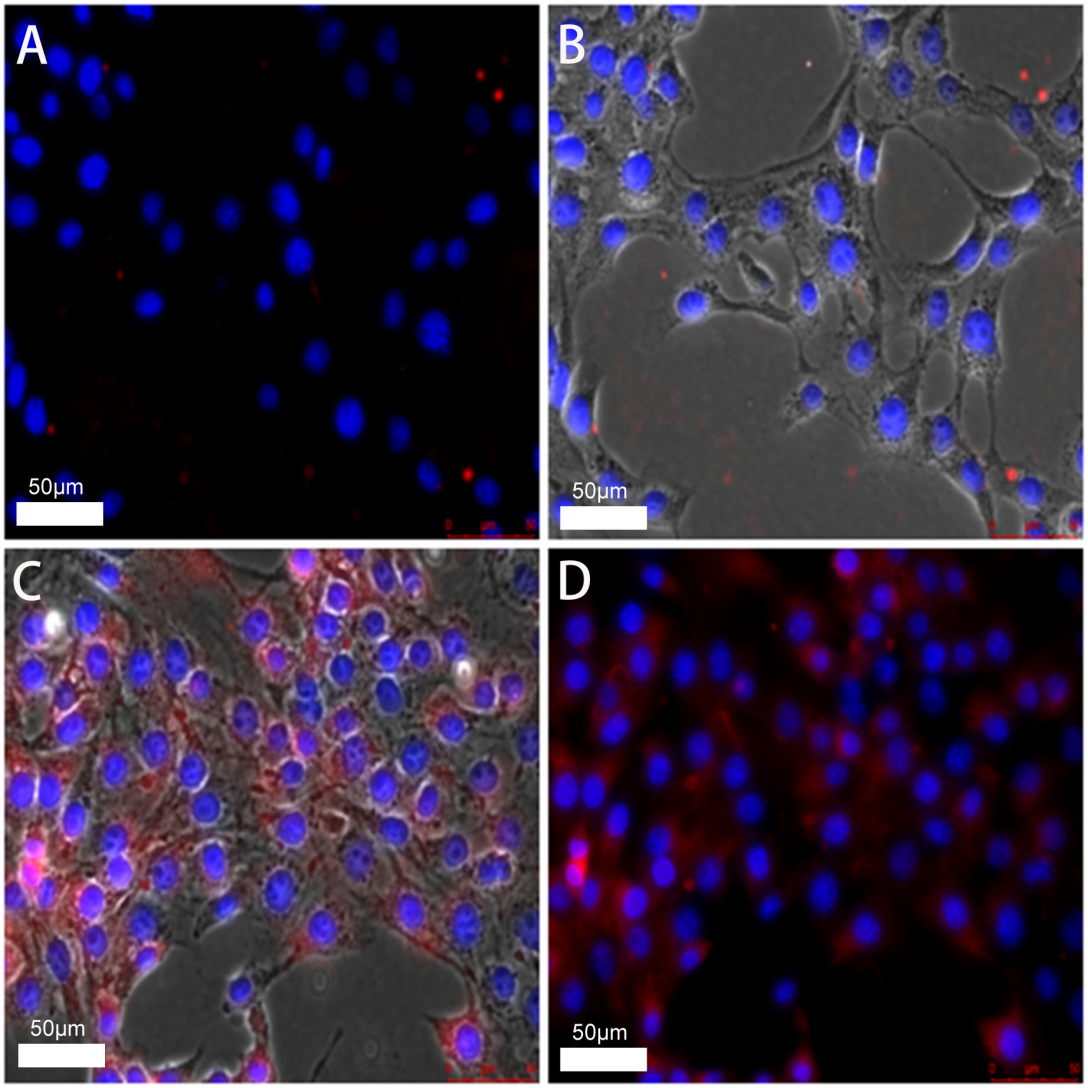
**(A, B)** confocal images of N-TNBs-DIL co-cultured with 4T1 cells after DAPI staining. **(C, D)** confocal images of FA-TNBs-DIL co-cultured with 4T1 cells after DAPI staining. It shows that TNBs is more strongly associated with breast cancer.

Under the confocal microscope, the cell nuclei were stained with DAPI staining solution. They showed a blue-violet color, whereas the DIL-loaded liposomes combined with the cell membrane dyed the cell membrane red. After rinsing with PBS solution, the red fluorescence in the FA-TNBs group was significantly greater than in the N-TNBs group, and a significant amount of red fluorescence was bound around the cells (**Figure 5**). Compared with the two groups, the red fluorescence intensity inside the FA-TNBs was also significant. This indicated that FA-TNBs could target and bind to the 4T1 cells, and the volume of microvesicles entering the cell interior was also higher. This was very beneficial for further imaging.

**Figure 6:**
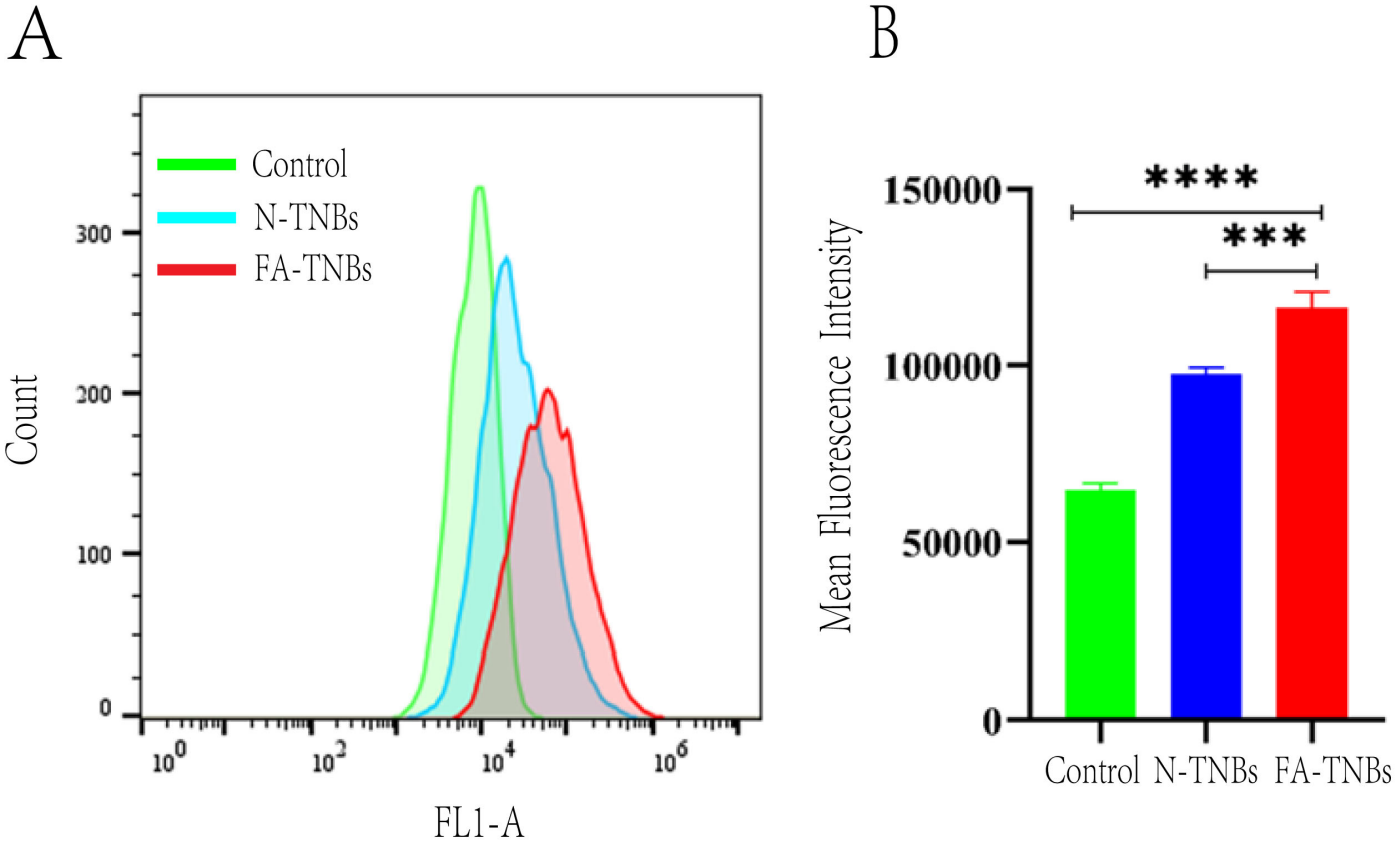
**(A)** In the blank group, after N-TNBs FITC and FA -TNBs FITC were co-cultured with 4T1 cells for 12 hours, 4T1 cells were targeted to the FITC fluorescence channel on flow cytometry. **(B)** In the blank group, after N-TNBs FITC and FA-TNBs FITC were co-cultured with 4T1 cells for 12 hours, the fluorescence intensity of nano microbubbles in their cells was measured by flow cytometry (n = 3, ***p < 0.001, **** p < 0.0001).

As seen from the flow cytometry data, the FA-TNBs group had higher channel targeting and FICT fluorescence values in cells than the N-TNBs group (**Figure 6**). This showed that compared with the NBS group, the FA-TNBs group had higher targeted binding to the 4T1 cells. Hence, the number of microvesicles entering the 4T1 cells was also higher.

### Cytotoxicity assay

**Figure 7:**
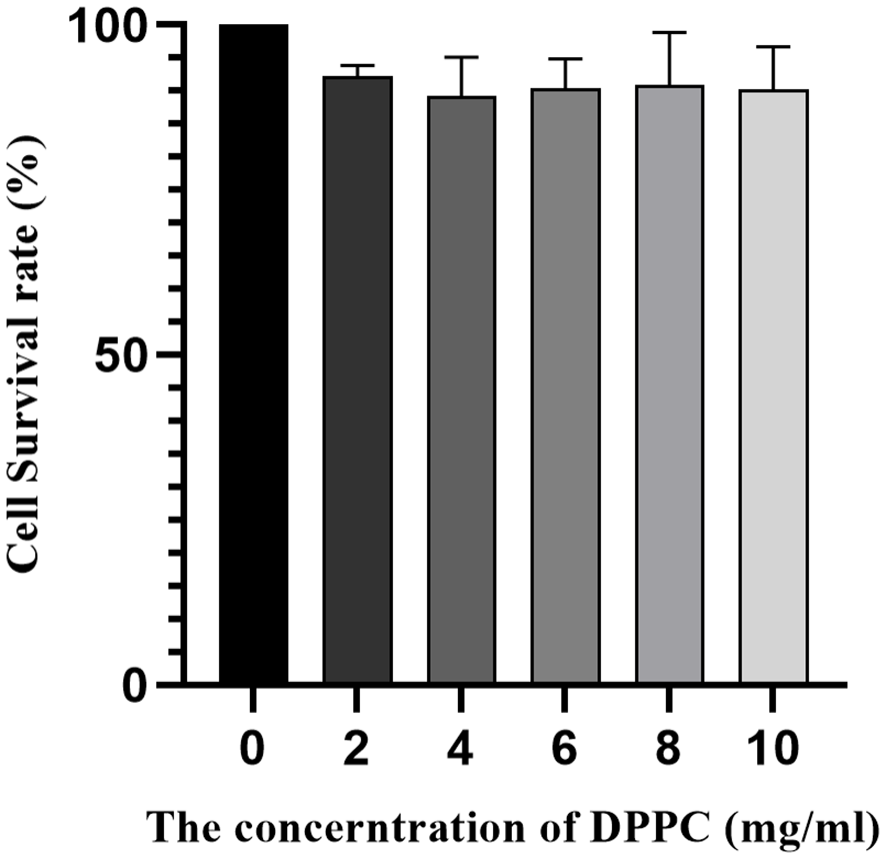
Survival rate of 4T1 cells at different concentrations of DPPC by FA-TNBs.

When the concentration of DDPC was 2 mg/mL, 4 mg/mL, 6 mg/mL, 8 mg/mL, and 10 mg/mL, there was no significant inhibitory effect on the growth of 4T1 cells. In this concentration range, cell viability was 90% greater (**Figure 7**). However, the DPPC concentration used for animal and *in vitro* imaging was less than 10 mg/mL. Therefore, the nanobubbles we prepared were of a material with better biocompatibility and had no significant effect on cell viability.

### *In vitro* imaging of nanobubbles

**Figure 8:**
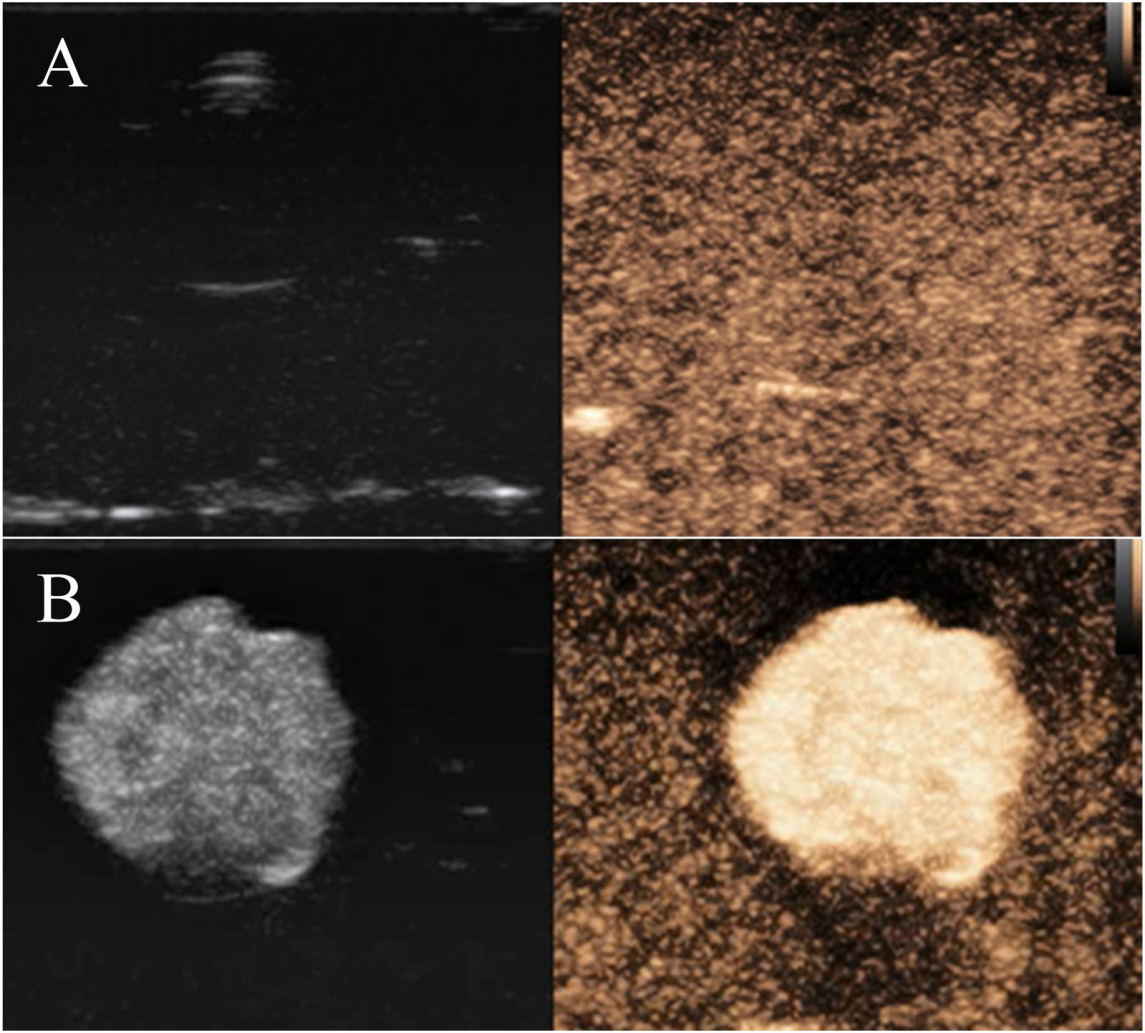
**(A)** Contrast-enhanced ultrasonography of PBS solution in agar block model; **(B)** Contrast-enhanced ultrasonography of FA-TNBs solution in the agar block model.

After injection of PBS buffer solution as a blank control, the imaging of nanobubbles showed no enhancement (**Figure 8**). In contrast, FA-TNBs showed enhanced performance (**Figure 8**). The time enhancement curve analysis demonstrated that the enhancement intensity of the PBS solution fluctuated around 20 dB and that of FA-TNBs around 44 dB. Compared with the PBS solution, the enhancement intensity of FA-TNBs was significantly higher. After five mins of observation, FA-TNBs still showed substantial enhancement, proving that the prepared nanobubbles had an excellent development effect and could be further used for *in vivo* experimental development.

### *In vivo* imaging

**Figure 9:**
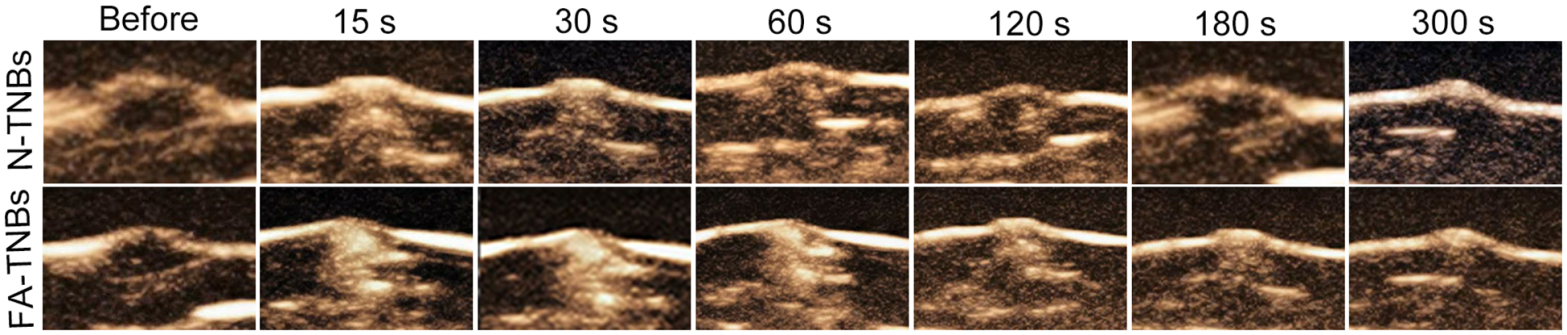
The contrast images at each period after injection of 200 ul of FA-TNBs or N-TNBs solution into the 5 mm tumor on the mice’s backs. It can be seen that after injection for 15s, both FA-TNBs and N-TNBs group mice showed high enhancement of tumor region, and wash-out appeared with the extension of contrast agent, and the wash-out time in FA-TNBS group was longer than that in N-TNBs group.

After intertumoral injection of FA-TNBs and N-TNBs, both showed angiographic solid findings, but the regression rate of FA-TNBs was slower than that of N-TNBs (**Figure 9**).

**Figure 10:**
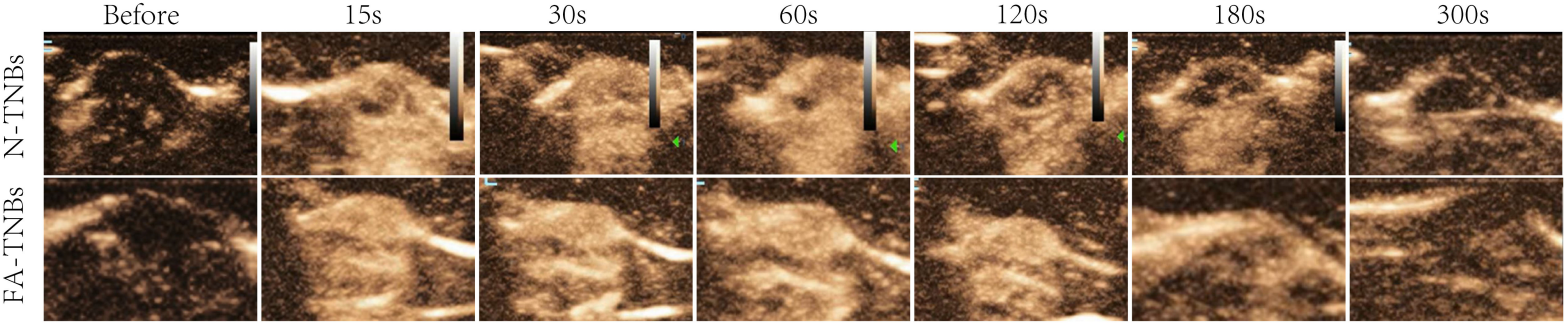
The contrast images of the 5 mm tumor on the mice’s backs at each time period after tail vein injection of 200 ul FA-TNBs and N-TNBs. After injection for 15s, both FA-TNBs and N-TNBs group mice showed high enhancement of tumor region, and the wash-out time in FA-TNBS group was longer than that in N-TNBs group.

After 200 ul of FA-TNBs and N-TNBs were injected into the tail vein (**Figure 10**), both began to enhance within 15 s of injection, but the contrast enhancement in the FA-TNBs group was significant. The FA-TNBs group peaked at about 20 s and the N-TNBs group at about 40 s. In addition, the peak intensity was 38.3 ± 1.5 dB in the FA-TNBs group (n = 3) and 31.7 ± 1.5 in the N-TNBs group dB (n = 3) (p < 0.05). At 120 s, the N-TNBs group showed significant regression performance, whereas the FA-TNBs group still had strong enhancement performance. At 180 s, the FA-TNBs group gradually began to subside, while most of the N-TNBs group had subsided (p < 0.05).

### Biosafety test results

**Figure 11:**
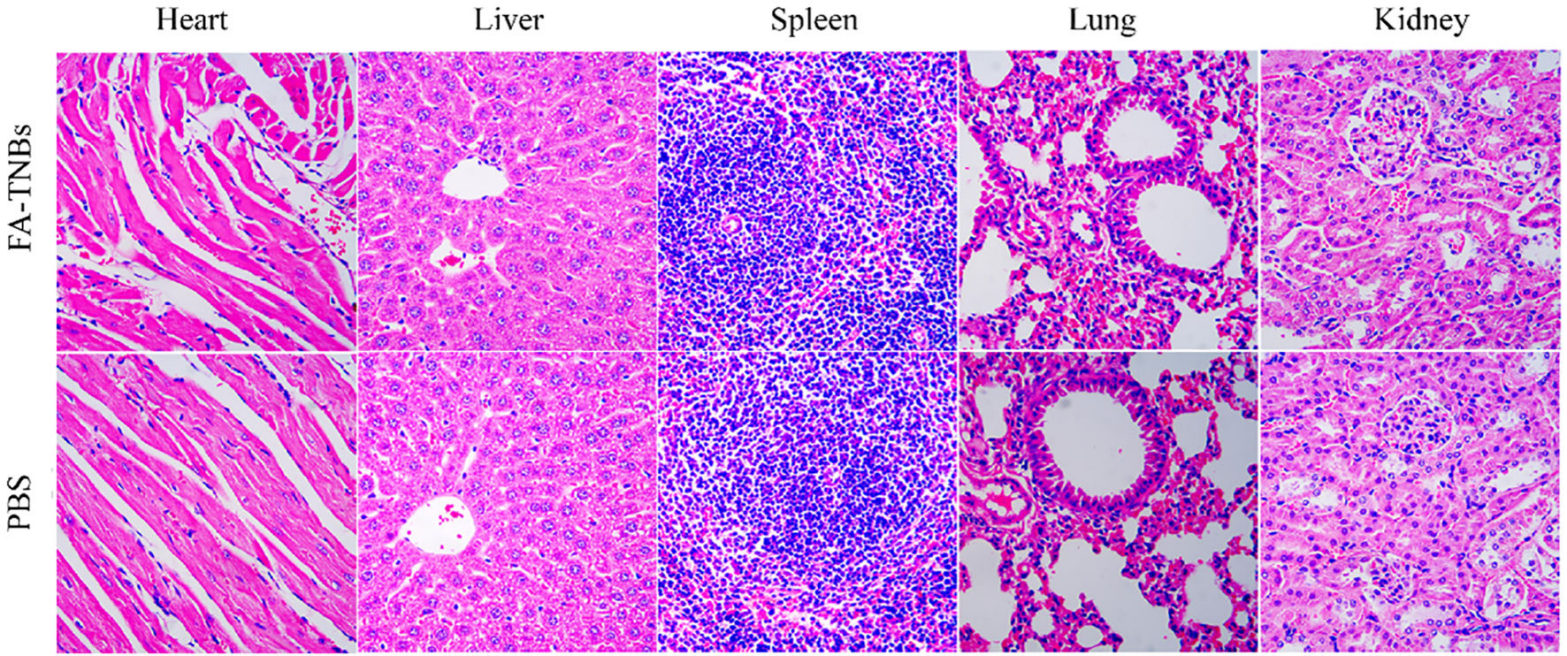
Important mouse tissue sections injected with 200 ul FA-TNBs after two weeks of natural life (200X).

**Figure 12:**
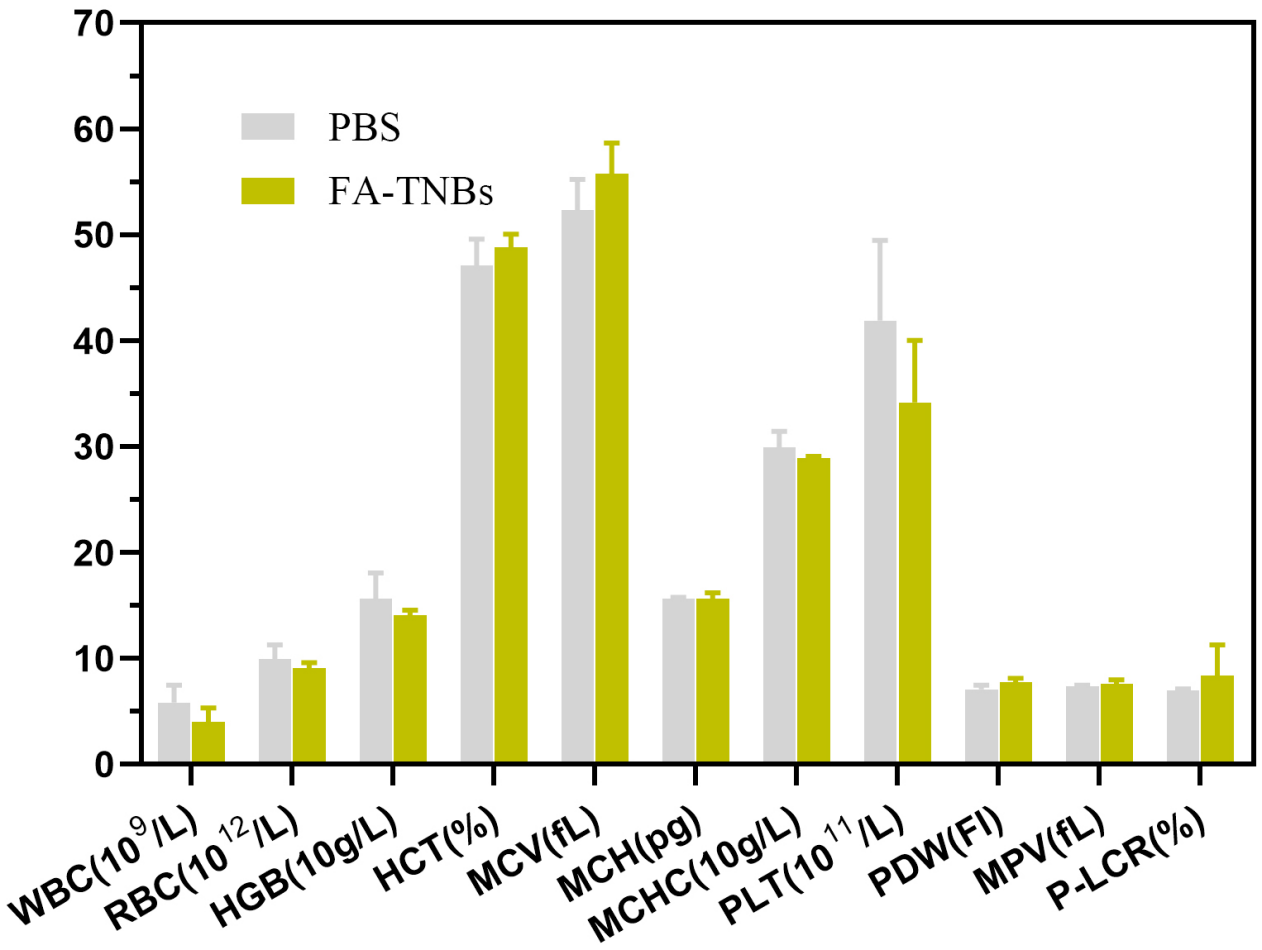
Hematological examination of mice injected with 200 ul FA-TNBs after two weeks of natural life.

Compared with the control group, there were no detectable pathological manifestations of tissue damage, such as necrosis, inflammation, hemorrhage, or pulmonary fibrosis (**Figure 11**). Except for PLT, there was no significant difference in routine blood between the control and FA-TNBs groups (**Figure 12**).

## Discussion

This study improved the process of preparing nanoliposomes by thin film hydration. In addition, we found that increasing the negative pressure more uniformly every few minutes during rotary evaporation and finally stabilizing at the negative target pressure contributed to smooth, uniform lipid film formation at the bottom of the eggplant-shaped flask. Hydration with water bath ultrasonic hydration followed by ultrasonic pulverization for an appropriate period can significantly reduce liposome particle size and PDI. The FA-TNBs particle size we prepared was 244 ± 21 nm, while the truncated diameter of the tumor neovascular gap was about 380–780 nm (13). Therefore, FA-TNBs should be able to pass through the tumor vascular gap and enter the tumor tissue.

CCK-8 experiments demonstrated that FA-TNBs did not inhibit the growth of 4T1 cells, proving they are safe, reliable, and biologically sound. The FA-TNBs group in the uptake experiment showed significantly higher intracellular fluorescent values and selectivity for the channels corresponding to the FITC fluorescent dye compared to the N-TNBs group, demonstrating the targeting of FA-TNBs to breast cancer 4T1 cells. At the same time, the entry into 4T1 cells showed a significant advantage, as represented by our FA-TNBs. This finding offers the possibility of a targeted diagnosis of microscopic breast cancer.

Our FA-TNBs had a similar vigorous contrast intensity to SonoVue (current microbubble contrast agents in clinical use) *in vitro* and lasted for several minutes. Both have significantly better contrast performance than the PBS solution. Although SonoVue is a micron-sized microbubble, the scattering ability of a single microbubble is more vital for ultrasound. The microbubble concentration of the nanoliposomal microbubble is much higher at a similar liposome concentration, so our targeted microbubble was slightly stronger than SonoVue in the overall *in vitro* contrast intensity, which suggested that nanoscale liposomal microbubbles had diagnostic value and potential in the clinic. Sheng D et al. studied the contribution of PFP vaporization caused by light irradiation to ultrasonic signals, which was specifically manifested as the strong and bright echo signal detected by the group containing PFP in contrast mode after laser irradiation, which played a role in achieving imaging. FA-TNBs prepared in this study has the function of direct development after injection, and has better angiographic effect than N-TNBs (14).

The biosafety of the microbubbles used *in vivo* must be guaranteed. Therefore, we injected a sufficient volume of microbubbles into the mice and verified the safety of the microbubbles using vital tissue sections and hematological assays. The imaging performance of the microbubbles *in vivo* was observed by intra-tumoral and tail vein injection in mice, respectively. We verified that our microbubbles had good imaging performance *in vivo* by both injection methods and that the targeting of FA-TNBs was significantly better than that of N-TNBs for tumors. In addition, *in vivo* experiments demonstrated the active targeting of our FA-TNBs to 5-mm mouse breast cancer tumors. Clinically, early breast cancer (tumors ≤5 mm) is associated with a higher survival rate compared to advanced disease and often allows for breast-conserving surgery. Although imaging techniques such as magnetic resonance imaging (MRI), mammography, and ultrasonography are crucial for breast cancer detection, accurately detecting tumors of 5 mm or smaller remains challenging with these conventional methods. Therefore, choosing a tumor size of approximately 5 mm in our *in vivo* imaging experiments reflects the clinical importance of early detection and aims to evaluate the sensitivity of FA-TNBs in identifying small tumors. In this study, biosafety evaluation was primarily conducted through hematological analysis and histological examination of major organs (heart, liver, spleen, lungs, and kidneys) to assess systemic toxicity and potential tissue damage. However, we acknowledge that other toxicological effects, such as inflammatory responses and immunogenicity, were not systematically evaluated in the current work. These aspects are critical for comprehensive preclinical safety assessment, and we plan to include cytokine profiling, immune cell activation markers, and inflammatory tissue markers in future studies to thoroughly investigate potential immune responses elicited by FA-TNBs. We emphasize that our FA-TNBs integrate targeted delivery, ultrasound contrast enhancement, and potential for early tumor detection in a single platform. Compared to conventional ultrasound contrast agents, our FA-TNBs demonstrate improved cellular uptake and imaging performance due to active targeting via the folate receptor, which is overexpressed in 4T1 breast cancer cells. We have also clarified the rationale for selecting the 4T1 murine breast cancer cell line, as it is a well-established, highly tumorigenic model that closely mimics the behavior of human triple-negative breast cancer in both growth and metastasis. Its high expression of folate receptors makes it an appropriate model for evaluating folate-targeted imaging systems. Furthermore, the choice of a liposome-based delivery system was based on several advantages: biocompatibility, structural stability, ease of functionalization with targeting ligands, and the ability to encapsulate gas for ultrasound contrast. These properties make liposome-derived nanobubbles particularly suitable for safe, targeted imaging *in vivo*.

Limited Tumor Models: Only the subcutaneous 4T1 tumor model was used. While this model is convenient, it does not fully replicate the tumor microenvironment of human breast cancer. Future studies should incorporate orthotopic and genetically engineered mouse models to better mimic the clinical scenario. 2) Short-Term Stability Evaluation: The stability of FA-TNBs was only assessed over a short period (24 hours). Long-term storage stability, circulation half-life, and degradation behavior in biological environments should be evaluated.3) Lack of Detailed Pharmacokinetics and Biodistribution: The study did not include comprehensive pharmacokinetic analysis or biodistribution studies. Radiolabeling or fluorescent tagging of FA-TNBs could help track their *in vivo* behavior over time. 4) Limited Toxicological Assessments: Only basic biosafety evaluations were performed. Additional assessments such as cytokine profiling, complement activation, and immunogenicity testing are needed to fully evaluate potential adverse immune responses.

Future Directions: 1) Development of orthotopic and spontaneous tumor models to test diagnostic accuracy and targeting efficacy in a more clinically relevant setting.2) Expanded toxicology and immunology panels to assess safety more comprehensively. 3) Functional studies to explore the therapeutic potential of FA-TNBs as drug or gene delivery carriers, enabling theranostic applications.

By addressing these limitations, future research can better establish the translational value of FA-TNBs for early breast cancer diagnosis and potentially for image-guided therapy.

In conclusion, the targeting and imaging effects of FA-TNBs in this study were verified through *in vitro* assays and cellular experiments. In addition, in mouse 5-mm tumor imaging, the FA-TNB group showed ultrasound imaging superiority compared with the N-TNBs group and had particular active targeting effects for tumor exactness. These findings are promising for the early diagnosis of microscopic breast cancer. The *in situ* experiment of mouse mammary carcinoma can be increased.

## Data Availability

The raw data supporting the conclusions of this article will be made available by the authors, without undue reservation.
